# Supplementary material for: Landmark knowledge overrides optic flow in honeybee waggle dance distance estimation
Source: J Exp Biol. 2024 Oct 23;227(21):jeb248162. doi: 10.1242/jeb.248162 (PMC11529883; doi:10.1242/jeb.248162)
Supplement: Supplementary information [file jexbio-227-248162-s1.pdf]

Table S1. Supplement 1: related to Fig. 4.
Data for each experiment (column 2: Exp1, Exp6, Exp7), average waggles (column 3), and average duration (column 4).

Available for download at
https://journals.biologists.com/jeb/article-lookup/doi/10.1242/jeb.248162#supplementary-data

Table S2. Supplement 2: related to Table 1.
Data for each experiment (Exp 1, 5, 6, 7); data show number of waggles for each experiment.

Available for download at
https://journals.biologists.com/jeb/article-lookup/doi/10.1242/jeb.248162#supplementary-data

Table S3. Supplement 3: related to Table 1.
Data for each experiment (Exp 1, 5, 6, 7); data show number of waggles for each experiment, separated for individual sessions.

Available for download at
https://journals.biologists.com/jeb/article-lookup/doi/10.1242/jeb.248162#supplementary-data
